# Antibiotic Resistance in Shiga Toxigenic *Escherichia coli* Isolates from Surface Waters and Sediments in a Mixed Use Urban Agricultural Landscape

**DOI:** 10.3390/antibiotics10030237

**Published:** 2021-02-26

**Authors:** Yvonne Ma, Jessica Chen, Karen Fong, Stephanie Nadya, Kevin Allen, Chad Laing, Kim Ziebell, Ed Topp, Laura M. Carroll, Martin Wiedmann, Pascal Delaquis, Siyun Wang

**Affiliations:** 1 Faculty of Land and Food Systems, University of British Columbia, Vancouver, BC V6T 1Z4, Canada; yvonne.ma@ubc.ca (Y.M.); jlc232@gmail.com (J.C.); karen.fong@ubc.ca (K.F.); stephanienadya@gmail.com (S.N.); kevin.allen@ubc.ca (K.A.); 2 National Centre for Animal Diseases, Canadian Food Inspection Agency, Lethbridge, AB T1J 3Z4, Canada; chad.laing@canada.ca; 3 Laboratory for Foodborne Zoonoses, Public Health Agency of Canada, Guelph, ON N1G 3W4, Canada; kim.ziebell@canada.ca; 4 London Research and Development Centre, Agriculture and Agri-Food Canada, London, ON N5V 4T3, Canada; ed.topp@canada.ca; 5 Department of Food Science, Cornell University, Ithaca, NY 14853, USA; laura.carroll@embl.de (L.M.C.); martin.wiedmann@cornell.edu (M.W.); 6 Summerland Research and Development Centre, Agriculture and Agri-Food Canada, Summerland, BC V0H 1Z0, Canada; pascal.delaquis@canada.ca

**Keywords:** Shiga toxin-producing *E. coli*, antimicrobial resistance, whole-genome sequencing, bacteriophages

## Abstract

Antibiotic resistance (AR) phenotypes and acquired resistance determinants (ARDs) detected by in silico analysis of genome sequences were examined in 55 Shiga toxin-producing *Escherichia coli* (STEC) isolates representing diverse serotypes recovered from surfaces waters and sediments in a mixed use urban/agricultural landscape in British Columbia, Canada. The isolates displayed decreased susceptibility to florfenicol (65.5%), chloramphenicol (7.3%), tetracycline (52.7%), ampicillin (49.1%), streptomycin (34.5%), kanamycin (20.0%), gentamycin (10.9%), amikacin (1.8%), amoxicillin/clavulanic acid (21.8%), ceftiofur (18.2%), ceftriaxone (3.6%), trimethoprim-sulfamethoxazole (12.7%), and cefoxitin (3.6%). All surface water and sediment isolates were susceptible to ciprofloxacin, nalidixic acid, ertapenem, imipenem and meropenem. Eight isolates (14.6%) were multidrug resistant. ARDs conferring resistance to phenicols (*floR*), trimethoprim (*dfrA*), sulfonamides (*sul1/2*), tetracyclines (*tetA/B*), and aminoglycosides (*aadA* and *aph*) were detected. Additionally, narrow-spectrum β-lactamase *bla*TEM-1b and extended-spectrum AmpC β-lactamase (cephalosporinase) *bla*CMY-2 were detected in the genomes, as were replicons from plasmid incompatibility groups IncFII, IncB/O/K/Z, IncQ1, IncX1, IncY and Col156. A comparison with surveillance data revealed that AR phenotypes and ARDs were comparable to those reported in generic *E. coli* from food animals. Aquatic environments in the region are potential reservoirs for the maintenance and transmission of antibiotic resistant STEC, associated ARDs and their plasmids.

## 1. Introduction

The Shiga toxigenic *Escherichia coli* (STEC) pathotype is characterized by the production of one or more cytotoxic Shiga-like toxins, small proteins encoded by a temperate lambdoid phage on the main chromosome. Infection with STEC causes diseases of variable severity, ranging from comparatively mild to acute hemorrhagic diarrhea, and potentially fatal thrombotic thrombocytopenic purpura or hemolytic uremic syndrome (HUS). Foodborne outbreaks have been reported with increasing frequency since two epidemiologically significant disease clusters were definitively associated with the consumption of red meat in the US in 1983 [1], and STEC are now recognized as significant water and foodborne threats to global public health [2,3,4]. A recent food-borne source attribution analysis performed using disease and surveillance data from several countries showed that foods from a broad range of categories are implicated in outbreaks [5]. Moreover, the analysis confirmed that plant-based foods, primarily fresh fruits and vegetables, are now significant vehicles for the foodborne transmission of STEC, notably in the Americas.

The advisability of antibiotic therapies for the management of STEC infections is the subject of enduring debate due to reports of antibiotic-induced toxin release from dead cells or by the induction of Shiga toxin-converting prophage [6]. For example, the fluoroquinolone antibiotic ciprofloxacin was shown to cause the induction of bacteriophage and Shiga toxin production in both in vivo and in vitro studies in a mouse model [7]. However, definitive evidence of causative association with clinical symptoms of human STEC disease remains elusive. A review of available data from clinical studies led Agger et al. [8] to conclude that it remains unclear whether the administration of protein and cell wall synthesis inhibitors influences the duration of diarrhea or the development of HUS. Moreover, a mixture of ciprofloxacin and the carbapenem meropenem was successfully used to alleviate the symptoms of HUS in patients infected with *E. coli* serotype O104:H4 during a major foodborne outbreak in Germany [9]. Nevertheless, antibiotic therapies are presently contraindicated by clinicians and public health authorities in some jurisdictions, primarily in the developed world. There is little doubt, however, that antibiotics are often administered to patients infected with STEC where diagnosis is delayed or masked by infections caused by other agents of gastrointestinal disease. Moreover, their use persists in many countries, including Canada, for the control and treatment of livestock diseases caused by STEC, notably edema disease in weaned piglets and watery diarrhea in calves [10].

The persistent therapeutic use of antibiotics in human and veterinary medicine or for growth promotion in animal production has contributed to the emergence and proliferation of antibiotic resistant bacteria (ARB), including zoonotic pathogenic species [11]. The global emergence of acquired resistance and concomitant decline in efficacy are anticipated to lessen the therapeutic value of present-day antibiotics used in human and veterinary medicine, with potentially dire social and economic consequences [12]. A report by the World Health Organization highlights the importance of resistance in *E. coli* due to the prevalence and wide spectrum of infections caused by variable pathotypes of the species [13]. Early clinical human STEC stains (primarily of serotype O157:H7) were generally susceptible to antibiotics, but there is evidence that antibiotic resistance (AR) is increasingly common in the pathotype [14,15,16,17,18,19]. Some isolates are reported to be multidrug resistant (MDR) according to the proposed standardized terminology for acquired resistance in Magiorakos et al. [20], whereby so-designated bacteria are not susceptible to more than one agent in more than three antimicrobial classes specified for the family *Enterobacteriaceae*. *E. coli* share many enzymatic and non-enzymatic resistance mechanisms with other species of Gram-negative bacteria, including intrinsic mechanisms involving inactivating enzymes or efflux pumps or mechanisms acquired by the horizontal transfer of antibiotic resistance determinants (ARDs) on mobile genetic elements leading, to the impaired absorption, modification of the antibiotic target by mutation or post-translational modification, or inactivation by hydrolysis or other chemical means [21]. Horizontal transfer is favoured by integrons, site-specific recombination systems consisting of two conserved segments flanking a central region containing “cassettes” of functional genes [22]. Integrons of relevance in the development of antibiotic resistance in enteric bacterial pathogens are primarily borne on self-replicating plasmids [23]. Most of the 27 recognized main incompatibility (Inc) groups of plasmids in *Enterobacteriaceae* have been associated with resistance to antibiotics in *E. coli*, including STEC [24,25,26]. 

Nucleic acid sequencing and in silico analysis are widely used to characterize ARB and associated ARDs in species recovered from disparate environments [27,28]. The current knowledge about AR and ARDs in STEC is largely based on the analysis of human clinical specimens or isolates obtained through surveillance systems that primarily target livestock species and meat products. In contrast, little is known about resident or transient STEC populations in natural or anthropogenically impacted terrestrial and aquatic ecosystems. The latter can serve as secondary reservoirs for transmission to humans by indirect routes, notably the consumption of food crops grown in environments susceptible to pollution from urban or agricultural sources [29]. The assessment of potential exposure risks and the development of mitigation measures require knowledge about the abundance and distribution of ARB and ARDs in such environments. In the present work, we report on AR phenotypes and associated ARDs in STEC recovered from surface waters in the Fraser Valley of British Columbia, Canada, an urban–agricultural region that supports intensive livestock farming and food crop production under irrigation.

## 2. Results

### 2.1. Antibiotic Resistance Phenotypes of STEC Isolates Recovered from Surface Water and Sediments

**Figure 1:**
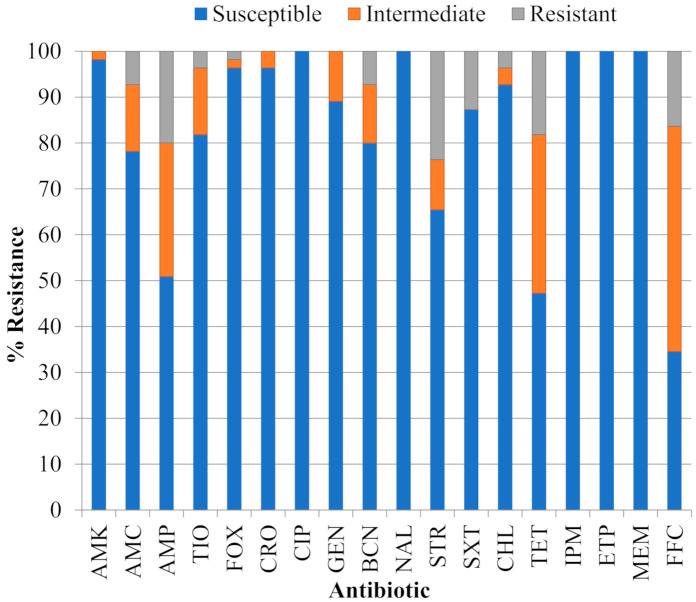
Proportion of 55 Shiga toxigenic *Escherichia coli* (STEC) isolates that were susceptible, of intermediate resistance or resistant to eighteen antibiotics: amikacin (AMK), amoxicillin/clavulanic acid (AMC), ampicillin (AMP), ceftiofur (TIO), cefoxitin (FOX), ceftriaxone (CRO), ciprofloxacin (CIP), gentamicin (GEN), kanamycin (BCN), nalidixic acid (NAL), streptomycin (STR), trimethoprim-sulfamethoxazole (SXT), chloramphenicol (CHL), tetracycline (TET), imipenem (IPM), ertapenem (ETP), meropenem (MEM) and florfenicol (FFC).

The proportion of 55 STEC isolates resistant, of intermediate resistance or susceptible to eighteen antibiotics is shown in Figure 1 and individual resistance phenotypes are provided in Table 1. All isolates were susceptible to the quinolone nalidixic acid (NAL), the fluoroquinolone ciprofloxacin (CIP) and carbapenems imipenem (IPM), ertapenem (ETP) and meropenem (MEM). The percentage of isolates resistant or of intermediate resistance to the other classes/groups of antibiotics examined were (in decreasing order of abundance): phenicols florfenicol (FFC), 65.5% and chloramphenicol (CHL), 7.3%; tetracycline (TET), 52.7%; penicillin ampicillin (AMP), 49.1%; aminoglycosides streptomycin (STR) 34.5%, kanamycin (BCN) 20.0%, gentamicin (GEN) 10.9%, and amikacin (AMK) 1.8%; penicillin + β-lactam inhibitor amoxicillin/clavulanic acid (AMC), 21.8%; third generation cephalosporins ceftiofur (TIO), 18.2% and ceftriaxone (CRO), 3.6%; folate pathway inhibitor trimethoprim-sulfamethoxazole (SXT), 12.7%; and the cephamycin cefoxitin (FOX), 3.6%. Resistance at concentrations examined in this work was limited to 10 of the antibiotics including FFC (16.4% of isolates), STR (23.6%), TET (18.2%), AMP (20.0%), SXT (12.7%), BCN (7.3%), AMC (7.3%), CHL (3.6%), TIO (3.6%) and FOX (1.8%). According to the proposed standardized terminology for acquired resistance profiles specified for the family *Enterobacteriaceae* in Magiorakos et al. [20], 10 isolates (18.2%) were pansusceptible, 37 (67.2%) were of intermediate resistance and eight (14.6%) were MDR. Four of the MDR isolates were from serogroup O111 and the most complex phenotype was found in isolate 385-O111:NM, which was resistant to nine antibiotics (AMP^R^ AMC^R^ CHL^R^ FFC^R^ FOX^R^ STR^R^ SXT^R^ TET^R^ TIO^R^) and showed intermediate resistance to two additional aminoglycosides (CRO^I^ GEN^I^) and one third generation cephalosporin (BCN^I^). 

### 2.2. Genomic Analysis of the STEC Isolates

A phylogeny inferred from the analysis of whole genome sequences was indicative of close relationships between isolates within analogous serogroups, as illustrated by the clustering of all O26:H11, O111:H8/NM and O157:H7 isolates from water/sediment and clinical sources (Figure 1). Exceptions were noted for some pairs (O109:H5/O117:H7, O5:NM/O165:H25, O116:H25/O188:H25, O91:H21/O76:H19) that clustered despite differences in serological assignment and are occasionally isolated from human, animal or environmental sources, but reports of human infections caused by strains from these serogroups are infrequent [30,31]. Few isolates have been studied in detail and little is known about their phylogenetic relationship to other serogroups. The sequences of some O gene clusters are known to differ by only a few nucleotides [32]. Hence, clustering of the isolates with different serovars by whole genome SNPs may reflect a true close evolutionary relatedness of isolates representing these different serovars. 

**Figure 2:**
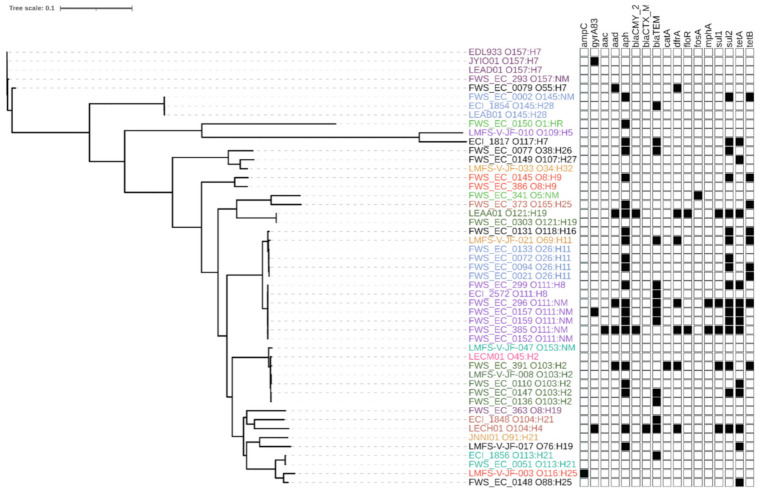
Maximum likelihood phylogeny constructed using core single nucleotide polymorphisms (SNPs) identified among the genomes of 55 STEC isolates recovered from water and sediments (isolate prefix FWS and LMFS) and 112 Canadian clinical STEC isolates and reference strain EDL933. Isolates of the same serotype with identical antimicrobial resistance (AMR) pattern or lacking AMR genes were omitted from the tree; tip colors denote different serotypes. Black squares in the matrix denote the presence of antibiotic resistance determinants common to the isolates. The scale corresponds to the number of substitutions per site. Resistance determinant functions: *ampC*: mutation in the promoter region of the chromosomal *ampC* gene; *gyrA*83 mutation: mutation in DNA gyrase codon 83; *aac*: aminoglycoside-3-acetyltransferase; *aadA*: aminoglycoside-3-adenylyltransferase; *aph*: aminoglycoside-3-phosphotransferase; *bla*CMY-2: AmpC β-lactamase; *bla*CTX-M: extended-spectrum β-lactamase; *bla*TEM: β-lactamase; *catA*: chloramphenicol acetyltransferase; *dfrA*: resistance to trimethoprim; *floR*: florfenicol/chloramphenicol exporter; *fosA*: fosfomycin thiol transferase; *mphA*: macrolide-2-phosphotransferase; *sul1*: dihydropteroate synthase; *sul2*: dihydropteroate synthase; *tetA*: tetracycline efflux protein; *tetB*: tetracycline efflux protein.

A presence–absence matrix displayed next to the tree in Figure 2 illustrates the array of ARDs detected in the genomes of water/sediment and clinical STEC isolates; the prevalence and distribution of specific genes are provided in Table 2. ARDs conferring resistance to phenicols (*catA, floR*) and fosfomycin (*fosA*) were found infrequently. ARDs that encode resistance to sulfonamides (*sul1/2*), trimethoprim (*dfrA*) tetracyclines (*tetA/B*) and aminoglycosides (*aac*, *aadA,* and *aph*) were more common, although the *aac* aminoglycoside-3-acetyltransferase gene was only detected in a single isolate from water. Narrow-spectrum β-lactamase *bla*_TEM-1b_ and extended-spectrum AmpC β-lactamase (cephalosporinase) *bla*_CMY-2_ -encoding gene sequences were also present in isolates from both sources, while the extended-spectrum β-lactamase-encoding *bla*_CTX-M_ and the chromosomal *gyrA83* mutation associated with quinolone/fluoroquinolone resistance were restricted to one and three clinical isolates, respectively. In addition to these acquired mechanisms of β-lactam resistance, three environmental isolates displayed mutations in the *ampC* promoter region. The chloramphenicol resistance effector *catA* was detected in one isolate from water. While macrolide resistance was not measured in the present study, the presence of the *mphA* ARD in two environmental isolates was noted in light of reports that suggest the prevalence of the gene is increasing in *E. coli* [33]. Overall, categorical analysis using Fisher’s exact and Bonferroni test indicated that there were no significant differences (*p* > 0.05) in the frequency of ARDs in isolates from either source (Table 2). 

The molecular basis of resistance was examined in more detail in the eight MDR isolates. ARDs with a known association to resistance to specific antibiotics are displayed adjacent to the resistance profiles in Table 3. Concordance was observed between phenotype determined in vitro and resistance predicted by in silico analysis in all isolates except 373-O165:H25, which was resistant to FFC despite our inability to detect known phenicol ARDs. The intermediate resistance in most isolates could be attributed to the presence of ARDs that impart reduced susceptibility to specific classes of antibiotics. However, none were detected in isolates with intermediate resistance to phenicols, in three isolates with intermediate resistance to the penicillins or penicillin + β-lactam inhibitors AMP and AMC, and one with intermediate resistance to the cephalosporin TIO. The plasmid replication sequences detected in each MDR isolate are also shown in Table 3. Replicons homologous to sequences in Inc incompatibility groups were detected in all the isolates: IncFII(pRSB107) was detected in three isolates, IncFIB replicons were detected in three isolates, IncFII(pHN7A8), IncFII, and IncQ1 were found in two isolates, and lastly IncX1 and IncY were each detected in one isolate. Replicons homologous to IncB/O/K/Z were common and were detected in six of eight isolates. ColE1 were identified in three isolates and Col156, a small colicin-encoding ColE-like plasmid which is often referred to as ColE(pIGJC156), was detected in two MDR isolates.

## 3. Discussion

Data on the prevalence of antibiotic resistance in STEC from either clinical or environmental sources in the province of British Columbia is scarce. Allen et al. [37] reported that 10 *E. coli* O157:H7 and 15 non- serotype O157 human clinical isolates were susceptible to the second-generation fluoroquinolone ciprofloxacin (CIP) and the carbapenem imipenem (IPM), antimicrobial drugs that are presently listed as Category I based on their very high importance in human medicine [38]. Maal-Bared et al. examined the resistance to six antibiotics in 27 *E. coli* O157:H7 isolates recovered from water, sediment and biofilms in a British Columbia watershed (Elk Creek) heavily impacted by agriculture and found that all were susceptible to CIP [39]. In the present study, 55 STEC isolates representing different serotypes and recovered from surface water and sediments in four watersheds located in the same region were also susceptible to CIP and the carbapenems IPM, ertapenem (ETP) and meropenem (MEM). Directed studies performed in different countries [40,41,42,43,44,45] and passive surveillance of laboratory-confirmed O157 STEC in the US [46] indicate that resistance to fluoroquinolones and carbapenems is historically rare in STEC from clinical, animal or food sources. The provincial public health authority (British Columbia Centre for Disease Control, BCCDC) compiles data and analyzes antibiotic resistance trends in clinical human *E. coli* isolates of all pathotypes captured by surveillance programs. A recent report was examined to assess the scope of resistance to Category I antibiotics in strains that cause human infections for comparison with rates measured in the environmental STEC isolates. In 2014, 23.8% of clinical *E. coli* isolates were resistant to CIP [47]. CIP is the third most widely prescribed antibiotic in Canada, primarily as a second-line drug for the treatment of persistent human infections (principally of the urinary tract). The response to drug pressure in community and hospital settings is likely contributing to increased resistance to this antibiotic in some human *E. coli* pathotypes [48,49]. While CIP is no longer licensed for veterinary purposes, use of other fluoroquinolones (eg., danofloxacin, enrofloxacin) persists, primarily for the control of respiratory diseases in cattle and pigs [50]. However, the susceptibility of environmental isolates suggests that the on-farm use of fluoroquinolones is not presently a significant driver of resistance in STEC. The incidence of carbapenamase-producing clinical strains also remains very low in the province [47]. Hence, the prudent use of “last-line agent” carbapenems in human medicine and their prohibition for the treatment, disease prevention and/or growth promotion of farm animals appears to be forestalling resistance to these antibiotics in *E. coli* from all pathotypes, including STEC. 

The susceptibility of environmental STEC to fluoroquinolones and carbapenems was reassuring in light of alarming rates of resistance reported in some jurisdictions. However, resistance or intermediate resistance to other Category I (penicillin-β-lactamase inhibitor combinations, third generation cephalosporins), high importance Category II (aminoglycosides, penicillins, cephamycins, folate pathway inhibitors) and to several classes of Category III antibiotics was recurrent in comparatively small collections of STEC isolates from the region. The comparison with provincial surveillance data indicated that rates of resistance to penicillins (AMP, 49.8%, 49.1%), aminoglycosides (GEN, 9.1%, 10.9%), folate pathway inhibitors (SXT, 24.0%, 12.7%) and third generation cephalosporins (cefotaxime, 11.2%, CRO, 3.6%) are similar in human clinical *E. coli* and environmental STEC isolates [47]. Provincial surveillance does not presently provide information about resistance to tetracyclines or phenicols in clinical *E. coli*. Both are administered to food-producing animals in Canada to prevent disease and for growth promotion. The Canadian Antimicrobial Resistance Surveillance System (CARSS) captures data on resistance to a range of antibiotics, including tetracyclines and phenicols, in generic *E. coli* isolated during production, slaughter and in retail meats derived from poultry, swine and cattle [51]. Because the surface waters sampled in this work are situated in a region of intensive livestock production, resistance rates in the environmental STEC were also examined against surveillance data derived from analysis of *E. coli* from meat animals and their products. Data from 2014 indicate that 55.3% of generic *E. coli* were resistant to TET, 32.2% to AMP, 29.7% to STR, 12.0% to STX and 8.1% to CRO [51]. Reciprocal resistance patterns in clinical [37] and environmental STEC described in previous work [39] and the present study suggests that common selection pressures, modes of transmission and mechanisms are likely driving resistance to antibiotics from these classes in the species. Unfortunately, none of the active surveillance systems capture data on phenicol resistance. CHL is prohibited from use in food producing animals but FFC, a fluorinated derivative of CHL, has a long history of use for the treatment of bovine respiratory diseases and to control pen disease in the local fish farming industry [52]. While 92.7% of STEC from water and sediments were susceptible to CHL, 16.4% were resistant and 49.1% were of intermediate resistance to FFC. Surprisingly, the overall prevalence of ARDs associated with resistance to CHL/FFC (*floR*, 1.20%) or CHL (*catA*, 0.60%) was very low. Moreover, none were detected in the MDR isolate 373-O165:H25 which was resistant to FFC. Consequently, the extent and molecular basis of resistance to phenicols in the STEC isolates could not be explained on the basis of the analyses performed in the present work and are clearly deserving of further investigation. 

In contrast, ARDs associated with resistance to tetracyclines (*tetA/B*), penicillins (*blaTEM*), aminoglycosides (*aac, aph*) and folate pathway inhibitors (*dfrA, sul1/2*) were detected in the sequences of environmental and clinical STEC at frequencies that were not significantly different (*p* > 0.05). Several studies have shown that *sul1/2, dfr, aac, aph, tetA*/*B* and *blaTEM* ARDs are widely distributed in *E. coli* from aquatic environments impacted by human activity [53,54,55,56]. The β-lactamase encoding *bla*TEM-1B was detected at the highest frequency in the present work, although two MDR isolates lacking the ARD were of intermediate resistance to AMP, possibly due to the presence of undetected or unknown efflux pumps which can lessen susceptibility to antibiotics [57,58]. The AmpC β-lactamase encoding *bla*CMY-2 ARD associated with resistance to third-generation cephalosporins is reported to be widespread in *E. coli* [59] but was only detected in one MDR isolate (385-O111-NM). Interestingly, this isolate displayed the most complex antibiophenotype and was resistant to both TIO and FFC. The *floR* gene is often situated on chromosomal DNA, although carriage of both *floR* and *bla*CMY-2 on an IncA/C plasmid has been reported [60,61]. IncA/C plamids were not detected in the isolates, but other incompatibility types previously shown to carry *bla*CMY-2 genes in *E. coli* from human and animal sources were found, including IncB/O and IncFII [24,62]. 

In conclusion, the analysis of STEC isolates from surface waters and sediments in a region of intensive agricultural activity revealed AR phenotypes and the presence of ARDs comparable to those reported in generic *E. coli* from food animals. This finding suggests that common selective pressures are contributing to the development of AR in commensal and pathogenic variants of the species, and that aquatic environments can serve as reservoirs for the maintenance of resistant STEC, associated ARDs and their plasmids. Hence, the use of surface waters for the irrigation of food crops could introduce the risk of exposure, particularly in commodities that are commonly consumed raw. 

## 4. Materials and Methods 

### 4.1. Bacterial Strains and Culture Conditions

STEC isolates (*n* = 55) from 21 different serotypes isolated from surface waters and sediments from four watersheds in a mixed use urban–agricultural landscape located in the Fraser Valley of British Columbia, Canada, by Nadya et al. [29] and Falardeau et al. [63] were examined in this study (Table S1). The isolates were recovered from sampling sites located upstream or downstream of water sources used for the irrigation of various crops, including berry fruits and market vegetables. Samplings were carried out monthly over two years. All isolates were confirmed to be STEC using a hydrophobic grid membrane immunoblot method that targets both Stx1 and Stx2, and the presence of the associated genes was confirmed by multiplex PCR [29,63]. *E. coli* strain ATCC® 25922™ was used as a positive control in antibiotic resistance assays (Cedarlane Labs, Burlington, ON, Canada). All strains were stored at −80 °C in tryptic soy broth (TSB; Becton, Dickson and Company, Sparks, MD, USA) with 20% glycerol. Working cultures were prepared by application of thawed cultures to the surface of tryptic Soy agar (TSA; Becton, Dickson and Company), followed by incubation at 37 °C for 24 h. Working cultures were held at 4 °C for a maximum of 30 days. 

### 4.2. Antibiotic Susceptibility Testing 

Susceptibility to antibiotics was determined by the Kirby–Bauer disc diffusion assay [64]. Briefly, 70 µL aliquots of 18–24 h cultures grown in Mueller–Hinton broth (Becton, Dickson and Company) at 37 °C were mixed with 7 mL of 0.75% molten agar (44 °C) and added to the surface of petri plates containing Mueller–Hinton agar (Becton, Dickson and Company). The plates were incubated at an ambient temperature for 10 min and antibiotic discs were applied to the surface of the agar. The zones of inhibition were measured to the nearest millimeter after incubation at 37 °C for 24 h. The assignment of the isolates to the “susceptible”, “intermediate resistance” and “resistant” categories was based on the diameter of the inhibition zone according to the Clinical and Laboratory Standards Institute (CLSI) guidelines [65,66,67]. *E. coli* ATCC® 25922™ was used as the reference strain to verify assay performance. Eighteen antibiotics from several classes or groups of antimicrobials used in animal and/or human medicine were tested, including: aminoglycosides (amikacin (AMK; 20/10 μg), gentamicin (GEN; 10 μg), kanamycin (BCN; 30 μg), streptomycin (STR; 10 μg)); phenicols (chloramphenicol (CHL; 30 μg) and florfenicol (FFC; 30 µg)); quinolones (nalidixic acid (NAL; 30 μg)); second generation fluoroquinolones (ciprofloxacin (CIP; 5 μg)); tetracyclines (tetracycline HCl (TET; 30 μg)); folate pathway inhibitors (trimethoprim-sulfamethoxazole (SXT)); penicillin + ß-lactamase inhibitors (amoxicillin/clavulanic acid (AMC; 30 μg)); penicillins (ampicillin (AMP; 10 μg)); cephamycins (cefoxitin (FOX; 30 μg)); third generation cephalosporins (ceftiofur (TIO; 30 μg), ceftriaxone (CRO; 30 µg)); and carbapenems (ertapenem (ETP; 10 µg), imipenem (IPM; 10 μg) and meropenem (MEM; 10 µg)). 

### 4.3. Genomic Analyses

The genomes of the STEC isolates from water/sediment were sequenced and assembled as described in Nadya et al. [31] and Fong et al. [68]. All sequences are available in NCBI’s Sequence Read Archive (SRA; http://www.ncbi.nlm.nih.gov/sra/ accessed on 5 January 2021) database under Bioproject Accessions PRJNA287560 and PRJNA649237 [69,70]. De novo assemblies were generated using shovill v. 1.0.94, using gsize 5000000 and mincov of 10% of the average genome coverage, as determined by the run_assembly_metrics.pl script in CG-pipeline (https://github.com/lskatz/CG-Pipeline accessed on 5 January 2021) [71]. An additional 112 assembled genomes from Canadian human clinical STEC isolates of variable serotypes from the same database were included in some of the analyses for comparative purposes. Tables S1 and S2 provide a list of accession numbers for each isolate, along with resistance genes, mutations and plasmids detected as described below. A single nucleotide polymorphism (SNP) phylogeny was generated using Parsnp v. 1.5.2 with *E. coli* O157:H7 EDL933 as a reference. The –x option to remove recombination regions was employed. An unrooted tree was visualized using Tree of Life [72]. Isolates of the same serotype with identical AMR pattern or lacking AMR determinants were omitted from the tree. Assemblies were queried for ARDs in the ResFinder database version from 28 October 2020, using staramr v. 0.4.0, with 90% nucleotide identity and 50% gene coverage cutoffs. Relevant mutations in the *ampC* promoter region, *gyrA, gyrB*, *parC*, and *parE* were detected by comparing to the PointFinder Database (https://bitbucket.org/genomicepidemiology/pointfinder_db/src/master/ accessed on 5 January 2021) using ARIBA v 2.12.0 where raw reads were available. For the clinical assemblies, the *ampC,* the *ampC* promoter, *gyrA, gyrB*, *parC*, and *parE* regions were extracted using a perl script (https://github.com/lskatz/lskScripts/blob/master/scripts/blastAndExtract.pl accessed on 5 January 2021), and subsequently aligned using MUSCLE in Geneious (version 6.0.6, Biomatters Ltd.). Relevant resistance conferring mutations were noted. Plasmid replicons were detected in each assembly using abricate 0.8.10 and a custom database based off the PlasmidFinder *Enterobacteriacae* database with a nucleotide identity cutoff of 90% and a coverage cutoff of 60% (database fasta available at https://github.com/StaPH-B/resistanceDetectionCDC/blob/master/plasmidDatabase.fasta accessed on 5 January 2021). Fisher’s exact test and Bonferroni correction to correct for multiple testing was calculated using R Studio (version 1.2.1335, http://www.rstudio.com/ accessed on 5 January 2021), and was used to determine if the prevalence of ARDs was non-randomly associated in water/sediment and clinical isolates [73]. 

## Figures and Tables

**Table 1:** Antibiotic resistance phenotypes of 55 STEC isolates from surface water and sediments in the Lower Mainland of British Columbia, Canada.

Isolate Number and Serotype	Antibiotic Resistance Phenotype ^1^	Source
Pansusceptible		
347-O174:H21	None	Water
005-O103:H2	None	Water
007-O103:H2	None	Water
008-O103:H2	None	Water
010-O109:H5	None	Water
033-O34:H32	None	Water
036-O34:H32	None	Water
039-O22:H8	None	Water
043-O153:NM	None	Water
047-O153:NM	None	Water
Intermediate resistant		
292-O177:NM	TET^I^ FFC^I^	Water
293-O157:NM	TIO^I^ GEN^I^ BCN^I^ STR^I^ TET^I^ FFC^I^	Water
294-O26:H11	TET^I^ FFC^I^	Water
300-O26:H11	FFC^I^	Water
301-O26:H11	FFC^I^	Water
337-O8:H19	TET^I^ FFC^I^	Water
338-O168:H8	FFC^R^ AMP^I^ TIO^I^ GEN^I^ BCN^I^ STR^I^ TET^I^	Water
340-O116:H25	AMC^R^ AMP^R^ AMK^I^ TIO^I^ FOX^I^ CRO^I^ GEN^I^ BCN^I^ STR^I^ TET^I^	Water
341-O5:NM	AMC^I^ AMP^I^ TET^I^ FFC^I^	Water
343-O98:NM	TIO^I^ GEN^I^ STR^I^ TET^I^ FFC^I^	Water
344-O5:NM	FFC^R^ AMC^I^ AMP^I^ TET^I^	Water
345-O22:H8	FFC^R^ AMC^I^ AMP^I^ TET^I^	Water
360-OR:H21	FFC^I^	Water
362-O5:NM	AMC^I^ FFC^I^	Water
363-O8:H19	TET^I^ FFC^I^	Water
365-O128:H2	AMP^I^ TET^I^ FFC^I^	Water
369-O98:NM	STR^I^ CHL^I^ TET^I^ FFC^I^	Water
370-O98:NM	CHL^I^ TET^I^ FFC^I^	Water
371-O157:H7	FFC^I^	Water
375-O157:H7	FFC^I^	Water
377-O103:H2	FFC^I^	Water
378-O103:H25	AMP^I^ TIO^I^ FFC^I^	Water
379-O26:H11	FFC^I^	Water
380-O165:NM	FFC^R^ AMP^I^ TET^I^	Water
381-O174:H8	STR^R^ AMP^I^ TIO^I^ GEN^I^ BCN^I^	Water
383-O163:H19	BCN^I^ STR^I^ TET^I^ FFC^I^	Water
384-O128:H2	AMP^I^ TET^I^	Water
386-O8:H9	AMP^I^ FFC^I^	Water
387-O103:H25	FFC^R^ AMP^I^	Water
389-O103:H2	AMP^I^ FFC^I^	Sediment
390-O103:H2	AMP^I^ FFC^I^	Sediment
003-O116:H25	AMX^R^, AMP^R^	Water
012-O116:H25	AMX^R^, AMP^R^	Water
017-O76:H19	STR^R^, TET^R^	Water
021-O69:H11	AMP^R^, STR^R^, SXT^R^, TET^R^	Water
025-O69:H11	AMP^R^, STR^R^, SXT^R^, TET^I^	Water
029-O69:H11	AMP^R^, STR^R^, SXT^R^, TET^I^	Water
Multidrug resistant		
296-O111:NM	AMP^R^ STR^R^ SXT^R^ TET^R^ AMC^I^ FFC^I^	Water
298-O111:NM	AMP^R^ BCN^R^ STR^R^ TET^R^ FFC^R^ AMC^I^ TIO^I^	Water
299-O111:H8	AMP^R^ TIO^R^ BCN^R^ STR^R^ TET^R^ AMC^I^ FFC^I^	Water
356-O69:H11	AMP^R^ STR^R^ SXT^R^ TET^R^ AMC^I^ FFC^I^	Water
373-O165:H25	BCN^R^ STR^R^ TET^R^ FFC^R^ AMP^I^ TIO^I^	Water
374-O165:NM	BCN^R^ STR^R^ TET^R^ FFC^R^ AMP^I^	Water
385-O111:NM	AMC^R^ AMP^R^ TIO^R^ FOX^R^ STR^R^ SXT^R^ CHL^R^ TET^R^ FFC^R^ CRO^I^ GEN^I^ BCN^I^	Water
391-O103:H2	STR^R^ SXT^R^ CHL^R^ TET^R^ AMP^I^ FFC^I^	Sediment

^1^ R denotes resistance and I intermediate resistance according to the Clinical and Laboratory Standards Institute (CLSI) guidelines. Antibiotics: amikacin (AMK), amoxicillin/clavulanic acid (AMC), ampicillin (AMP), cefoxitin (FOX), ceftiofur (TIO), ceftriaxone (CRO), chloramphenicol (CHL), ciprofloxacin (CIP), ertapenem (ETP), gentamicin (GEN), imipenem (IPM), kanamycin (BCN), meropenem (MEM), nalidixic acid (NAL), streptomycin (STR), tetracycline (TET) and trimethoprim-sulfamethoxazole (SXT) and florfenicol (FFC).

**Table 2:** Distribution of acquired resistance genes detected in whole genome sequences of 55 STEC isolates recovered from water and sediments in the Lower Mainland of British Columbia, Canada and 112 Canadian clinical STEC isolates. Probabilities that the prevalence of specific genes were nonrandomly distributed were calculated using Fisher’s exact test. Resistance determinant functions: *ampC*: mutation in the promoter region of the chromosomal *ampC* gene; *gyrA83*: mutation in DNA gyrase codon 83; *aac*: aminoglycoside 3-N-acetyltransferase; *aadA*: aminoglycoside-3-adenylyltransferase; *aph*: aminoglycoside-3-phosphotransferase; *bla*CMY-2: β-lactamase; *bla*CTX-M: extended spectrum β-lactamase; *bla*TEM: β-lactamase; *catA*: chloramphenicol acetyltransferase; *dfrA*: resistance to trimethoprim; *floR*: florfenicol/chloramphenicol exporter; *fosA*: fosfomycin thiol transferase; *mphA*: macrolide-2-phosphotransferase; *sul1*: dihydropteroate synthase; *sul2*: dihydropteroate synthase; *tetA*: tetracycline efflux protein; *tetB*: tetracycline efflux protein.

	Prevalence (%) in All Isolates	Water/Sediment Isolates		Clinical Isolates		*p* Value Fisher’s Extract	Bonferroni Corrected *p* Value
**Gene**		(+)	(−)	(+)	(−)		
*ampC*	1.80	3	52	0	112	0.034	0.585
*gyrA*83	1.80	0	55	3	109	0.552	1.000
*aac*	0.60	1	54	0	112	0.329	1.000
*aadA*	32.99	3	52	2	110	0.333	1.000
*aph*	16.17	12	43	15	97	0.184	1.000
*bla*CMY-2	1.80	1	54	2	110	1.000	1.000
*bla*CTX-M	0.60	0	55	1	111	1.000	1.000
*bla*TEM	12.57	7	48	14	98	1.000	1.000
*catA*	0.60	1	54	0	112	0.329	1.000
*dfrA*	5.99	7	48	3	109	0.015	0.263
*floR*	1.20	1	54	1	111	0.552	1.000
*fosA*	2.40	3	52	1	111	0.105	1.000
*mphA*	1.20	2	53	0	112	0.107	1.000
*sul1*	2.99	3	52	2	110	0.333	1.000
*sul2*	13.17	9	46	13	99	0.466	1.000
*tetA*	8.98	5	50	10	102	1.000	1.000
*tetB*	7.78	8	47	5	107	0.031	0.531

**Table 3:** Acquired resistance determinants (ARDs) and plasmid replicon sequences detected in whole-genome sequences of eight multidrug resistant (MDR) STEC isolates from surface water and sediments.

Isolate	Antibiotic Class/Group and Resistance Profile	ARDs Detected in the Sequences and Putative Role in Resistance ^1^	Plasmid Replicon Sequences ^2,3^
296-O111:NM	Aminoglycosides STR^R^ Folate pathway inhibitors SXT^R^ Penicillins, penicillin +β-lactam inhibitors AMP^R^ AMC^I^ Phenicols FFC^I^ Tetracyclines TET^R^ NT ^4^	*aadA2, aph(3″)-Ib, aph(6)-Id* *drfA8, dfrA12, sul1, sul2* *bla*TEM-1B *-* *tetA, tetB* *mphA*	ColE1 (*2*), IncB/O/K/Z, IncFII(pHN7A8), IncX1
298-O111:NM	Aminoglycosides BCN^R^ STR^R^ Cephalosporins TIO^I^ Penicillins, penicillin +β-lactam inhibitors AMP^R^ AMC^I^ Phenicols FFC^R^ Tetracyclines TET^R^ NT ^4^	*aph(3′)-Ia, aph(3″*)*-Ib, aph(6)-Id* *bla*TEM-1B *bla*TEM-1B *-* *tetA* *sul2*	Col156, IncB/O/K/Z, IncFII(pRSB107), IncQ1
299-O111:H8	Aminoglycosides BCN^R^ STR^R^ Cephalosprins TIO^R^ Penicillins, penicillin +β-lactam inhibitors AMP^R^ AMC^I^ Phenicols FFC^I^ Tetracyclines TET^R^ NT ^4^	*aph(3′)-Ia, s aph(3″)-Ib, aph(6)-Id* *bla*TEM-1B *bla*TEM-1B *-* *tetA* *sul2*	Col156, IncB/O/K/Z, IncFII(pRSB107), IncQ1
356-O69:H11	Aminoglycosides STR^R^ Folate pathway inhibitors SXT^R^ Penicillins penicillin +β-lactam inhibitors AMP^R^ AMC^I^ Phenicols FFC^I^ Tetracyclines TET^R^	*aph(3″)-Ib, aph(6)-Id* *dfrA8, sul2* *bla*TEM-1B *-* *tetB*	IncB/O/K/Z, IncFII(pHN7A8), IncFIB(AP001918)
373-O165:H25	Aminoglycosides BCN^R^ STR^R^ Cephalosporins TIO^I^ Penicillins AMP^I^ Phenicols FFC^R^ Tetracyclines TET^R^	*aph(3′)-1a, aph(3* *″* *)-Ib, aph(6)-Id* *-* *-* *-* *tetB*	IncFII, IncFIB(AP001918)
374-O165:NM	Aminoglycosides BCN^R^ STR^R^ Phenicols FFC^R^ Penicillins AMP^I^ Tetracyclines TET^R^	*aph(3′)-Ia, aph(3* *″* *)-Ib,aph(6)-Id* *-* *-* *tetB*	IncFII, IncFIB(AP001918),
385-O111:NM	Aminoglycosides STR^R^ BCN^I^ GEN^I^ Cephalosporins TIO^R^ CRO^I^ Cephamycins FOX^R^ Penicillins, penicillin + β-lactam inhibitors AMP^R^ AMC^R^ Folate pathway inhibitors SXT^R^ Phenicols CHL^R^ FFC^R^ Tetracyclines TET^R^ NT	*aac(3)-VIa, aadA1, aadA2, aph(3″*)-*Ib*, *aph(6)-Id* *bla*CMY-2 *bla*CMY-2 *dfrA12, sul1, sul2* *floR* *tetA* *mphA*	ColEI, IncB/O/K/Z, IncFII(pRSB107), IncY
391-O103:H2	Aminoglycosides STR^R^ Folate pathway inhibitors SXT^R^ Phenicols CHL^R^ FFC^I^ Penicillin AMP^I^ Tetracyclines TET^R^	*aadA1, aph(3* *″* *)-Ib, aph(6)-Id* *dfrA1, sul1, sul2* *catA1* *-* *tetB*	ColEI, IncB/O/K/Z

^1^ Resistance gene function: *aac(3)-VIa*: aminoglycoside 3-N-acetyltransferase; *aadA1*: aminoglycoside-3′-adenylyltransferase; *aadA2*: aminoglycoside-3-adenylyltransferase; *aph(3′)-Ia*: aminoglycoside-3- phosphotransferase; *aph(3″)-1b*: aminoglycoside-3-phosphotransferase; *aph(6)-Id*: aminoglycoside-3-phosphotransferase; *bla*TEM-1B: β-lactamase; *bla*CMY-2: AmpC β-lactamase-encoding β-lactamase; *catA1*: chloramphenicol acetyltransferase; *dfrA1*: resistance to trimethoprim; *drfA8*: resistance to trimethoprim; *dfrA12*: resistance to trimethoprim; *floR*: florfenicol/chloramphenicol exporter; *mphA*: macrolide-2-phosphotransferase, *aph(3″)-Ib*: streptomycin phosphotransferase; *aph(6)-Id*: streptomycin phosphotransferase; *sul1*: dihydropteroate synthase; *sul2*: dihydropteroate synthase; *tetA*: tetracycline efflux protein; *tetB*: tetracycline efflux protein. Some genes are listed multiple times where resistance to drug classes may be induced by the same gene. ^2^ Italicized parentheses denote the number of times the sequence was detected in the genome. ^3^ Predicted incompatibility group. Plasmid descriptions and putative role: ColEI: small plasmid, colicin-encoding. Col156: small plasmid, colicin-encoding [24,34]; IncB/O/K/Z: MDR and virulence genes [24]; IncFIB(AP001918): MDR and virulence genes [34]; IncFII(pRSB107): MDR and virulence genes [34]; IncFII(pHN7A8): MDR and virulence genes [35]; IncQ1: very broad host range plasmid, MDR genes [34]; IncX1: narrow host range conjugative plasmid, MDR and virulence genes [34,36]; IncY: Phage-like plasmid [34].^4^ NT: not tested.

## Data Availability

All data derived from the study are provided in the article and in supplementary material.

## Supplementary Materials

The following are available online at https://www.mdpi.com/2079-6382/10/3/237/s1, Table S1: serotypes, IDs, accession numbers for genomic sequence reads and assemblies, resistance genes, resistance mutations and plasmids identified in Shiga toxigenic *Escherichia coli* isolates recovered from surface waters and sediments in the Lower Mainland of British Columbia between 2012–2016, Table S2: serotypes, IDs, accession numbers for genomic sequence reads and assemblies, resistance genes, resistance mutations and plasmids identified in Shiga toxigenic *Escherichia coli* clinical isolates used for comparison of antibiotic resistance gene frequencies.
