# Supplementary material for: Antibiotic Resistance in Shiga Toxigenic Escherichia coli Isolates from Surface Waters and Sediments in a Mixed Use Urban Agricultural Landscape
Source: Antibiotics (Basel). 2021 Feb 26;10(3):237. doi: 10.3390/antibiotics10030237 (PMC7996769; doi:10.3390/antibiotics10030237)
Supplement: Supplementary file 1 [file antibiotics-10-00237-s001.pdf]

Table S1. Serotypes, IDs, accession numbers for genomic sequence reads and assemblies, resistance genes, resistance mutations and plasmids identified in Shiga toxigenic *Escherichia coli* isolates recovered from surface waters and sediments in the Lower Mainland of British Columbia between 2012-2016.

**Strain ID**	**Nomenclature used in manuscript**	**Accession No.**	**Resistance Genes**	**Resistance Mutations**	**Plasmids**
FWS_EC_347	347-O174:H21	SAMN08797219	None	None	IncFIB, IncB/O/K/Z
FWS_EC_292	292-O177:NM	SAMN08797175	None	None	IncFIC(FII), IncFIB
FWS_EC_293	293-O157:NM	SAMN08797176	None	None	IncFIA, IncFIB
FWS_EC_294	294-O26:H11	SAMN08797177	None	None	IncB/O/K/Z, ColE1, ColE1, IncFIB
FWS_EC_300	300-O26:H11	SAMN08797183	None	None	IncB/O/K/Z, IncB/O/K/Z, ColE1, ColE1, ColE1, IncFIB
FWS_EC_301	301-O26:H11	SAMN08797184	None	None	IncB/O/K/Z, IncB/O/K/Z, ColE1, ColE1, ColE1, IncFIB
FWS_EC_337	337-O8:H19	SAMN08797209	None	None	IncFIB
FWS_EC_338	338-O168:H8	SAMN08797210	None	None	IncFIB
FWS_EC_340	340-O116:H25	SAMN08797212	None	*ampC*_promoter_size_53bp(C12T)	IncFIC(FII), IncFIA, IncFIB, ColE1
FWS_EC_341	341-O5:NM	SAMN08797213	*fosA7*	None	IncFIB, ColE1, ColE1
FWS_EC_343	343-O98:NM	SAMN08797215	None	None	IncFII(pSE11), ColE1
FWS_EC_344	344-O5:NM	SAMN08797216	*fosA7*	None	ColE1, ColE1, IncFIB
FWS_EC_345	345-O22:H8	SAMN08797217	None	None	None
FWS_EC_360	360-OR:H21	SAMN08797232	None	None	IncB/O/K/Z, IncFIB
FWS_EC_362	362-O5:NM	SAMN08797234	*fosA7*	None	ColE1, ColE1, IncFIB
FWS_EC_363	363-O8:H19	SAMN08797235	None	None	IncI2(delta), IncFIB, Col156
FWS_EC_365	365-O128:H2	SAMN08797237	None	None	IncFII, IncFIB, ColE1, Col(MG828)
FWS_EC_369	369-O98:NM	SAMN08797241	None	None	p0111, IncFII(pSE11), ColE1
FWS_EC_370	370-O98:NM	SAMN08797242	None	None	p0111, IncFII(pSE11), ColE1
FWS_EC_371	371-O157:H7	SAMN08797243	None	None	IncFIB, ColE1
FWS_EC_375	375-O157:H7	SAMN08797247	None	None	IncFIB
FWS_EC_377	377-O103:H2	SAMN08797249	None	None	IncFIB

FWS_EC_378	378-O103:H25	SAMN08797250	None	None	p0111, IncFIB, Col156
FWS_EC_379	379-O26:H11	SAMN08797251	None	None	p0111, IncB/O/K/Z, ColE1, IncFIB
FWS_EC_380	380-O165:NM	SAMN08797252	None	None	IncFIB, IncFII(29)
FWS_EC_381	381-O174:H8	SAMN08797253	None	None	IncFII(29), IncB/O/K/Z, Col156, Col(MG828)
FWS_EC_383	383-O163:H19	SAMN08797255	None	None	IncI(pO113), IncFIB, ColE1, Col156
FWS_EC_384	384-O128:H2	SAMN08797256	None	None	IncFIB, IncFII
FWS_EC_386	386-O8:H9	SAMN08797258	None	None	IncY, IncFIA, IncFIB
FWS_EC_387	387-O103:H25	SAMN08797259	None	None	IncFIB
FWS_EC_389	389-O103:H2	SAMN08797261	None	None	IncB/O/K/Z, IncB/O/K/Z, IncFIB
FWS_EC_390	390-O103:H2	SAMN08797262	None	None	IncB/O/K/Z, IncFIB
FWS_EC_296	296-O111:NM	SAMN08797179	*aadA2*, *aph(3'')-Ib*, *aph(6)-Id*, *bla*TEM-1B, *dfrA12*, *dfrA8*, *mph(A)*, *sul1*, *sul2*, *tet(A)*, *tet(B)*	None	IncB/O/K/Z, IncFII(pHN7A8), IncX1, ColE1
FWS_EC_298	298-O111:NM	SAMN08797181	*aph(3'')-Ib*, *aph(3')-Ia*, *aph(6)-Id*, *bla*TEM-1B, *sul2*, *tet(A)*	None	IncB/O/K/Z, IncQ1, Col156, IncFII(pRSB107)
FWS_EC_299	299-O111:H8	SAMN08797182	*aph(3'')-Ib*, *aph(3')-Ia*, *aph(6)-Id*, *bla*TEM-1B, *sul2*, *tet(A)*	None	IncB/O/K/Z, IncQ1, Col156, IncFII(pRSB107)
FWS_EC_356	356-O69:H11	SAMN08797228	*aph(3'')-Ib*, *aph(6)-Id*, *bla*TEM-1B, *dfrA8*, *sul2*, *tet(B)*	None	IncFII(pHN7A8), IncB/O/K/Z, IncFIB
FWS_EC_373	373-O165:H25	SAMN08797245	*aph(3'')-Ib*, *aph(3')-Ia*, *aph(6)-Id*, *tet(B)*	None	IncFII, IncFIB
FWS_EC_374	374-O165:NM	SAMN08797246	*aph(3'')-Ib*, *aph(3')-Ia*, *aph(6)-Id*, *tet(B)*	None	IncFII, IncFIB
FWS_EC_385	385-O111:NM	SAMN08797257	*aac(3)-VIa*, *aadA1*, *aadA2*, *aph(3'')-Ib*, *aph(6)-Id*, *bla*CMY-2, *dfrA12*, *floR*, *mph(A)*, *sul1*, *sul2*, *tet(A)*	None	IncB/O/K/Z, IncY, IncFII(pRSB107), ColE1
FWS_EC_391	391-O103:H2	SAMN08797263	*aadA1*, *aph(3'')-Ib*, *aph(6)-Id*, *catA1*, *dfrA1*, *sul1*, *sul2*, *tet(B)*	None	IncB/O/K/Z, ColE1
LMFS-V-JF-003	003-O116:H25	SAMN15660548	None	*ampC*_promoter_size_53bp(C12T)	IncFIB, IncFIA, IncFIC(FII)
LMFS-V-JF-005	005-O103:H2	SAMN15660549	None	None	p0111, IncFIB
LMFS-V-JF-007	007-O103:H2	SAMN15660550	None	None	None

LMFS-V-JF-008	008-O103:H2	SAMN15660551	None	None	IncB/O/K/Z, IncFIB
LMFS-V-JF-010	010-O109:H5	SAMN15660552	None	None	IncFIB
LMFS-V-JF-012	012-O116:H25	SAMN15660553	None	*ampC*_promoter_size_53bp(C12T)	IncFIC(FII), IncFIB, IncFIA, ColE1
LMFS-V-JF-017	017-O76:H19	SAMN15660554	*aph(3'')-Ib*, *aph(6)-Id*, *tet(A)*	None	IncI1-I(gamma), ColE1, IncFIB, Col(MG828), IncFII(pCoo)
LMFS-V-JF-021	021-O69:H11	SAMN15660555	*aph(3'')-Ib*, *aph(6)-Id*, *bla*TEM-1B, *dfrA8*, *sul2*, *tet(B)*	None	IncFII(pHN7A8), IncB/O/K/Z, ColE1
LMFS-V-JF-025	025-O69:H11	SAMN15660556	*aph(3'')-Ib*, *aph(6)-Id*, *bla*TEM-1B, *dfrA8*, *sul2*, *tet(B)*	None	IncFII(pHN7A8), IncB/O/K/Z, ColE1, IncFIB
LMFS-V-JF-029	029-O69:H11	SAMN15660557	*aph(3'')-Ib*, *aph(6)-Id*, *bla*TEM-1B, *dfrA8*, *sul2*, *tet(B)*	None	IncFII(pHN7A8), IncB/O/K/Z, ColE1, IncFIB
LMFS-V-JF-033	033-O34:H32	SAMN15660558	None	None	IncFIB, Col(pHAD28)
LMFS-V-JF-036	036-O34:H32	SAMN15660559	None	None	IncFIB, Col(pHAD28)
LMFS-V-JF-039	039-O22:H8	SAMN15660560	None	None	IncFII(pHN7A8), IncFIB, ColE1
LMFS-V-JF-043	043-O153:NM	SAMN15660561	None	None	IncFII(pCoo), IncFIB, IncFII(pSFO)
LMFS-V-JF-047	047-O153:NM	SAMN15660562	None	None	IncFII(pSFO), IncFIB, IncFII(pCoo)

Table S2. Serotypes, IDs, accession numbers for genomic sequence reads and assemblies, resistance genes, resistance mutations and plasmids identified in Shiga toxigenic *Escherichia coli* clinical isolates used for comparison of antibiotic resistance gene frequencies.

Strain ID	Serotype	Accession no.	Resistance Genes	Resistance Mutations	Plasmids
FWS_EC_0001	O26:H11	SAMN08768102	None	None	ColE1, ColE1, IncFIB, p0111, IncB/O/K/Z
FWS_EC_0002	O145:NM	SAMN08768103	*aph(3")-Ib*, *aph(6)-Id*, *sul2*, *tet(B)*	None	ColpVC, IncFIB, IncB/O/K/Z
FWS_EC_0003	O45:H2	SAMN08768104	None	None	IncFIB
FWS_EC_0005	O111:NM	SAMN08768106	None	None	None
FWS_EC_0006	O121:H19	SAMN08768107	None	None	IncFIB, IncB/O/K/Z
FWS_EC_0007	O103:H2	SAMN08768108	None	None	IncFIB
FWS_EC_0008	O91:H21	SAMN08768109	None	None	IncFIB
FWS_EC_0010	O113:H21	SAMN08768111	None	None	Col(MP18), IncFIB, IncI(pO113), ColE1, ColE3
FWS_EC_0021	O26:H11	SAMN08796970	*tet(B)*	None	ColE2, IncFIB, ColE1, Col(MG828), p0111, IncB/O/K/Z, IncFII
FWS_EC_0024	O26:H11	SAMN08796973	None	None	ColE1, IncFIB, IncB/O/K/Z
FWS_EC_0026	O103:H2	SAMN08796975	None	None	IncFIB
FWS_EC_0032	O45:H2	SAMN08796981	None	None	IncFIB
FWS_EC_0051	O113:H21	SAMN08796998	None	None	IncFIB
FWS_EC_0070	O121:NM	SAMN08797016	None	None	None
FWS_EC_0071	O156:NM	SAMN08797017	None	None	IncFIB
FWS_EC_0072	O26:H11	SAMN08797018	*aph(3")-Ib*, *aph(6)-Id*, *sul2*	None	Col156, ColE1, Col(pHAD28), p0111, ColE1, IncFIB, IncB/O/K/Z
FWS_EC_0074	O75:H8	SAMN08797020	None	None	ColpVC, IncB/O/K/Z, IncFIB, IncFII(pCoo), ColE3, ColE1

FWS_EC_0075	O26:H11	SAMN08797021	None	None	ColE2, IncB/O/K/Z, IncFIB
FWS_EC_0076	O1:H20	SAMN08797022	None	None	IncI2(delta), IncFIB
FWS_EC_0077	O38:H26	SAMN08797023	*aph(3'')-Ib*, *aph(6)-Id*, *bla*TEM-1C, *sul2*	None	Col(MG828), IncFII(pCoo), ColE1, IncFIB, IncB/O/K/Z, Col156
FWS_EC_0078	O51:H49	SAMN08797024	None	None	ColE1, ColE1, IncI1-I(gamma), IncFIB, IncFII(pHN7A8)
FWS_EC_0079	O55:H7	SAMN08797025	aadA1, dfrA1	None	IncI2(delta), IncFIB
FWS_EC_0080	O119:H25	SAMN08797026	None	None	IncFIB
FWS_EC_0081	OR:NM	SAMN08797027	None	None	IncFIB, IncI2(delta), IncFIC(FII)
FWS_EC_0082	O21:H8	SAMN08797028	None	None	Col440II, Col(pHAD28), IncFIA(HI1), IncFIB
FWS_EC_0083	O28ab:NM	SAMN08797029	None	None	ColE1
FWS_EC_0084	O26:H11	SAMN08797030	None	None	Col(pHAD28), Col(pHAD28), ColE1, IncFIB, IncB/O/K/Z, ColE1, ColE1
FWS_EC_0085	O157:H16	SAMN08797031	None	None	IncFIB
FWS_EC_0094	O26:H11	SAMN08797040	*aph(3'')-Ib*, *aph(6)-Id*, *sul2*, *tet(B)*	None	IncFIB, IncFII(29), IncB/O/K/Z, Col(pHAD28), ColE1
FWS_EC_0100	O26:H11	SAMN08797046	None	None	ColE1, IncFIB, IncB/O/K/Z
FWS_EC_0101	O8:H16	SAMN08797047	None	None	IncFIB, Col156
FWS_EC_0102	O98:H29	SAMN08797048	None	None	ColE1, IncFII(pSE11)
FWS_EC_0103	O165:NM	SAMN08797049	None	None	IncFIB
FWS_EC_0104	O26:H11	SAMN08797050	None	None	IncFIB, IncB/O/K/Z, IncFII, Col156, ColE1
FWS_EC_0109	O26:NM	SAMN08797055	None	None	ColE1, IncFIB, IncB/O/K/Z
FWS_EC_0110	O103:H2	SAMN08797056	*aph(3')-Ia*, *tet(A)*	None	Col156, IncB/O/K/Z, IncFIB, IncB/O/K/Z
FWS_EC_0113	O103:H2	SAMN08797059	None	None	IncFIB, IncB/O/K/Z

FWS_EC_0128	O183:H21	SAMN08797074	None	None	IncB/O/K/Z, IncFIB
FWS_EC_0129	O71:H11	SAMN08797075	None	None	ColE1, IncFIB, IncB/O/K/Z
FWS_EC_0130	O182:H48	SAMN08797076	None	None	IncFIB
FWS_EC_0131	O118:H16	SAMN08797077	*aph(3")-Ib*, *aph(6)-Id*, *sul2*, *tet(B)*	None	IncFIB, IncB/O/K/Z
FWS_EC_0132	O177:NM	SAMN08797078	None	None	IncFIB, IncFIC(FII)
FWS_EC_0133	O26:H11	SAMN08797079	None	None	Col(pHAD28), IncFIB, IncB/O/K/Z
FWS_EC_0134	O6:NM	SAMN08797080	None	None	None
FWS_EC_0135	O49:NM	SAMN08797081	None	None	IncB/O/K/Z, IncFIB
FWS_EC_0136	O103:H2	SAMN08797082	*bla*TEM-1A	None	IncB/O/K/Z, IncFIB, Col156
FWS_EC_0137	O103:H2	SAMN08797083	None	None	IncFIB
FWS_EC_0138	O103:H2	SAMN08797084	None	None	IncI2(delta), IncFIB
FWS_EC_0139	O26:H21	SAMN08797085	None	None	ColE1, IncFIB, p0111, IncB/O/K/Z
FWS_EC_0140	O121:H1	SAMN08797086	None	None	IncFIB, IncB/O/K/Z
FWS_EC_0141	O69:H11	SAMN08797087	None	None	IncFIB, IncY, IncB/O/K/Z
FWS_EC_0142	O26:H21	SAMN08797088	None	None	ColE1, IncFIB, IncB/O/K/Z
FWS_EC_0143	O103:H2	SAMN08797089	None	None	IncFIB, IncB/O/K/Z
FWS_EC_0144	O26:H11	SAMN08797090	None	None	Col156, ColE1, ColE1, ColE1, IncFIB, IncB/O/K/Z
FWS_EC_0145	O8:H9	SAMN08797091	*aph(3")-Ib*, *aph(6)-Id*, *sul2*, *tet(B)*	None	IncY, IncFIB
FWS_EC_0146	O103:H25	SAMN08797092	None	None	IncFIB, Col156, IncI1-I(gamma), IncX4
FWS_EC_0147	O103:H2	SAMN08797093	*aph(3")-Ib*, *aph(3')-Ia*, *aph(6)-Id*, *bla*TEM-1B, *sul2*, *tet(A)*	None	IncB/O/K/Z, IncFIB, IncQ1

FWS_EC_0148	O88:H25	SAMN08797094	*tet(A)*	None	p0111, IncFIC(FII), IncFIB, IncFIA, IncI1-I(gamma), ColE1
FWS_EC_0149	O107:H27	SAMN08797095	*tet(A)*	None	pEC4115, IncI1-I(gamma), IncFIB, Col(pHAD28)
FWS_EC_0150	O1:HR	SAMN08797096	*aph(3'')-Ib*, *aph(6)-Id*	None	IncFIA, IncFIB, IncX1, Col(MG828)
FWS_EC_0151	O111:NM	SAMN08797097	*aph(3'')-Ib*, *aph(3')-Ia*, *aph(6)-Id*, *bla*TEM-1B, *sul2*, *tet(A)*	None	Col156, ColE1, IncFII(pRSB107), Col156, Col(MG828), IncB/O/K/Z, IncQ1
FWS_EC_0152	O111:NM	SAMN08797098	None	None	IncFII(pRSB107), ColE1, IncI2(delta), Col156
FWS_EC_0153	O121:H19	SAMN08797099	None	None	IncFIB, IncB/O/K/Z
FWS_EC_0154	O121:H19	SAMN08797100	None	None	IncFIB, IncB/O/K/Z
FWS_EC_0155	O121:H19	SAMN08797101	None	None	IncFIB, IncB/O/K/Z
FWS_EC_0156	O121:H19	SAMN08797102	*bla*CMY-2	None	IncFIB, IncI1-I(gamma), IncB/O/K/Z
FWS_EC_0157	O111:NM	SAMN08797103	*aph(3'')-Ib*, *aph(3')-Ia*, *aph(6)-Id*, *bla*TEM-1B, *sul2*, *tet(A)*	*gyrA*(S83L)	IncFII(pRSB107), ColE1, Col156, IncQ1, IncB/O/K/Z, Col156
FWS_EC_0159	O111:NM	SAMN08797105	*aph(3'')-Ib*, *aph(3')-Ia*, *aph(6)-Id*, *bla*TEM-1B, *sul2*, *tet(A)*	None	Col156, IncFII(pRSB107), Col(MG828), IncX4, p0111, Col156, IncQ1, IncB/O/K/Z
FWS_EC_0160	O121:H19	SAMN08797106	None	None	IncFIB, IncB/O/K/Z
FWS_EC_0302	O121:H19	SAMN08797185	None	None	IncFIB, IncI2(delta), IncB/O/K/Z, ColpVC
FWS_EC_0303	O121:H19	SAMN08797186	None	None	IncFIB, IncI2(delta), IncB/O/K/Z
FWS_EC_0346	O22:H8	SAMN08797218	None	None	None
JNNI01	O91:H21	SAMN02744162	None	None	IncFIB
JNNJ01	O91:H21	SAMN02744546	None	None	IncFIB
JYIO01	O157:H7	SAMN03342207	None	*gyrA*(S83L)	ColE1, IncFIB
LDYN01	O26:H11	SAMN03393401	None	None	None
LDZZ01	O121:H19	SAMN03393409	None	None	IncFIB, IncB/O/K/Z

LEAA01	O121:H19	SAMN03393410	*aadA2*, *aph(3'')-Ib*, *aph(6)-Id*, *bla*CMY-2, *dfrA12*, *floR*, *sul1*, *sul2*, *tet(A)*	None	IncC
LEAB01	O145:H28	SAMN03393411	None	None	IncFIB, IncB/O/K/Z
LEAD01	O157:H7	SAMN03393393	None	None	IncFIB
LEAK01	O157:H7	SAMN03393400	None	None	IncFIB
LECH01	O104:H4	SAMN03393405	*aph(3'')-Ib*, *aph(6)-Id*, *blaCTX-M-15*, *bla*TEM-1B, *bla*TEM-1B, *bla*TEM-1B, *dfrA7*, *sul1*, *sul2*, *tet(A)*	*gyrA*(S83A)	IncI1-I(gamma), Col(MG828), IncQ1, ColE1
LECM01	O45:H2	SAMN03393402	None	None	IncFIB
EDL933	O157:H7	SAMN02905113	None	None	None
ECI_1756	O157:H7	*	None	None	IncFIB, ColE1
ECI_1757	O157:H7	*	None	None	IncFIB, ColE1
ECI_1817	O117:H7	*	*aph(3'')-Ib*, *aph(6)-Id*, *bla*TEM-1B, *sul2*, *tet(A)*	None	Col(BS512), Col(BS512), Col(BS512), ColE1, Col(BS512), IncFIB, IncFII, IncB/O/K/Z, Col(BS512), IncFII(pRSB107)
ECI_1839	O91:H21	*	None	None	IncFII(pHN7A8), IncFIB
ECI_1848	O104:H21	*	*bla*TEM-116	None	IncB/O/K/Z, IncFIB, ColE1
ECI_1853	O103:H2	*	*bla*TEM-116	None	ColE1, IncFIB
ECI_1854	O145:H28	*	*bla*TEM-116	None	ColE1, IncFIB, IncB/O/K/Z
ECI_1856	O113:H21	*	*bla*TEM-116	None	IncFIB, ColE1
ECI_1858	O91:H21	*	None	None	IncFIB
ECI_1862	O111:H8	*	*bla*TEM-116	None	Col156, ColE1, IncFII(pRSB107)
ECI_1871	O145:H28	*	None	None	IncB/O/K/Z, IncFIB

ECI_2572	O111:H8	*	*bla*TEM-116	None	Col156, Col156, Col156, ColE1, Col156, Col156, Col156, Col156, Col156, IncFII(pRSB107)
ECI_2577	O111:H8	*	None	None	None
ECI_2600	O113:H21	*	None	None	IncFIA
ECI_2626	O26:H11	*	None	None	Col156, Col(pHAD28), Col(pHAD28), Col(pHAD28), ColE1, p0111, IncFIB, Col(pHAD28), Col(pHAD28), Col(pHAD28), Col(pHAD28), Col(pHAD28), ColE1, IncB/O/K/Z
ECI_2849	O136:H16	*	None	None	IncFIB, ColE1
ECI_2850	O5:NM	*	*fosA7*	None	IncFIB, Col(MG828), IncY, ColE1
ECI_2851	O26:H11	*	None	None	IncFIB, IncB/O/K/Z, ColE1
ECI_2852	O136:H46	*	None	None	ColE1, IncFII(pCoo), IncI1-I(gamma), IncX1, IncFIB, ColE1, ColE1, Col(MG828)
ECI_2853	O130:H11	*	None	None	IncFIB, IncI(pO113), Col156
ECI_2854	O118:H16	*	None	None	IncB/O/K/Z, IncFIB
ECI_2855	O111:NM	*	None	None	ColE1, IncFIB, ColE1
ECI_2856	O165:H25	*	None	None	IncFIB
ECI_2857	O165:NM	*	None	None	None
ECI_2858	O109:H5	*	None	None	None
ECI_2859	O73:H2	*	None	None	Col(MG828), IncFIB, ColE1, IncFII(pRSB107)
ECI_2860	O139:H19	*	None	None	IncI(pO113), IncFIB, ColE1
ECI_2861	O69:H11	*	None	None	IncFIB, IncB/O/K/Z
ECI_2862	OR:H29	*	None	None	None

*Confidential isolates from clinical sources.
